# Colorectal polyp risk is linked to an elevated level of homocysteine

**DOI:** 10.1042/BSR20171699

**Published:** 2018-04-20

**Authors:** Manchun Sun, Manyi Sun, Li Zhang, Songli Shi

**Affiliations:** 1 Department of Pharmacy, Zhongnan Hospital of Wuhan University, Wuhan University, Wuhan 430071, People’s Republic of China; 2 Department of Gastroenterology, Tianjin Union Medical Center, Tianjin 300121, People’s Republic of China; 3 Department of Pathology, Tianjin Union Medical Center, Tianjin 300121, People’s Republic of China

**Keywords:** colorectal polyps, folate, homocysteine, vitamin B12

## Abstract

Several studies have reported an association between levels of folate, homocysteine, and vitamin B_12_ and the risk of colorectal polyps. Here, our aim is to examine the possible effect of folate, homocysteine, and vitamin B_12_ levels on the risk of colorectal polyps by means of meta-analysis based quantitative synthesis. According to our inclusion/exclusion criteria, a total of 13 case–control studies were enrolled. The *P*-value of the association test, standard mean difference (SMD), and 95% confidence interval (CI) were calculated. Pooled analysis data showed a negative correlation between the risk of colorectal polyps and the levels of serum folate, red blood cell (RBC) folate, or vitamin B_12_ (all *P*>0.05). Nevertheless, for homocysteine level, we also observed a statistically significant difference between cases and controls in the overall and subgroup analysis of hospital-based control (HB), population-based control (PB), Chinese, Caucasian, or Asian (all *P*<0.05, SMD > 0). We found that increased levels of homocysteine may be statistically and significantly related to the risk of colorectal polyps.

## Background

Colorectal polyps are abnormal growths and protrusions on the colorectal surface [1,2]. The many types of colorectal polyps are classified based on their pathological properties like hyperplastic polyps and adenomatous polyps [3,4]. Hyperplastic polyps have less malignant potential than adenomatous polyps [4]. Although colorectal polyps are considered benign lesions, the malignant transformation of certain polyps, like sessile serrated colorectal polyps, is implicated in the carcinogenic process of the colon and rectum [5]. Endoscopic and laparoscopic surgery can be used to remove colorectal polyps [2,6,7]. Environmental factors, such as cigarette smoking or alcohol consumption, and genetic background may contribute to the initiation or development of colorectal polyps [8,9].

Folate, a water-soluble vitamin, is essential to various biochemical processes of cells, such as cell cycles or nucleic acid synthesis [10]. Homocysteine is linked to one-carbon transfer reaction, the adequate level of SAM and normal DNA methylation reactions [11]. Vitamin B_12_, also termed as cobalamin, has been found to be involved in homocysteine metabolic reactions, DNA synthesis, mitochondrial metabolism, and semen quality [12,13]. Folate, homocysteine, and vitamin B_12_ take part in the transmethylation process, which allows the transfer of methyl groups to specific substrates [14]. Folate deficiency and abnormal folate metabolic pathways are involved in the incidence of DNA hypomethylation or uracil misincorporation, and pathogenesis of several clinical diseases, such as reproductive abnormalities or colorectal cancer (CRC) [10,15,16]. In addition, low folate and low vitamin B_12_ status is correlated with an elevated homocysteine concentration, namely hyperhomocysteinemia, which is associated with several clinical diseases, mostly cardiovascular disorders [14,17].

There is no consensus regarding the correlation between the level of three methyl group donors (folate, homocysteine, and vitamin B_12_) and the risk of colorectal polyps [15,18–29]. Here, we first conducted a meta-analysis to examine this association using currently available data. The biochemical variables of red blood cell (RBC) folate, serum folate, homocysteine, and vitamin B_12_ were analyzed in cases of colorectal polyps and controls without polyps.

## Materials and methods

### Publication searching

We gathered relevant publications by extensive search of three online databases, including PubMed, WOS (Web of Science), and Embase (Excerpta Medica Database) through August 2017. Here, we list the term of PubMed database searching: ((((((((((((polyps [MeSH terms]) OR polyp) OR polyposis) OR colorectal polyps) OR colorectal polyp) OR colorectal polyposis) OR adenomatous polyposis) OR hyperplastic polyps) OR intestinal polyps) OR colonic polyps) OR polyposis coli) AND (((((((((folate) OR serum folate) OR RBC folate) OR red blood cell folate) OR folate status) OR folate metabolism)) OR (((((((homocysteine [MeSH terms]) OR 2-amino-4-mercaptobutyric acid) OR 2 amino 4 mercaptobutyric acid) OR homocysteine, L-isomer) OR homocysteine, L Isomer) OR L-Isomer homocysteine) OR plasma homocysteine)) OR ((((((((((vitamin B 12[MeSH terms]) OR B 12, vitamin) OR vitamin B12) OR B12, vitamin) OR cyanocobalamin) OR cobalamins) OR cobalamin) OR Eritron) OR VB12) OR serum vitamin B12)).

### Study screening

We then independently reviewed and screened the eligible case–control studies using our selection criteria, which were duplicate data; reviews; cases or trials; cell or animal data; meeting abstract or poster; meta-analysis; study of polyps other than colorectal, no mention of folate, homocysteine, or vitamin B_12_ levels; data of intake of folate or vitamin B_12_; lack of control data or S.D. The process of database searching and study selection was performed in accordance with the recommendations regarding preferred reporting items for systematic reviews and meta-analyses (PRISMA) [30].

### Data extraction

Next, we performed the data extraction based on the included original case–control studies. A specifically designed table was used to show the detailed characteristics of all studies, including first author name, year of publication, group, number of case–control studies, mean value, S.D. value, race, country, disease type, control source. Missing information is designated ‘NA’ (not available).

### Quantitative synthesis

Standard mean difference (SMD) was used as the evaluation criterion of the continuous data with varied measurement units, as in similar meta-analyses [31–33]. Base on Cohen statistics, *P*-value of association test, pooled SMD, and 95% confidence interval (CI) were synthesized in the overall and subgroup meta-analysis by such factors as race, country, and control source. A two-tailed *P*<0.05 was considered indicative of statistically significant difference. Additionally, based on Q-statistic and *I^2^* test, the evaluation of interstudy heterogeneity was carried out. *P*-values of Q-statistic < 0.05 or *I^2^* values > 50% were considered indicative of high heterogeneity. We here used an inverse variance (IV)-weighted, random-effects model.

### Publication bias

Both Begg’s test and Egger’s test were performed to quantitatively judge the possible publication bias. *P*-value of Begg’s test and Egger’s test < 0.05, and asymmetric funnel plot indicate significant publication bias.

### Sensitivity analysis

We performed a sensitivity analysis to evaluate the sources of heterogeneity and stability of the data. We removed each case–control study from the analysis one by one and analyzed differences in pooled data. All these tests were performed using STATA software (Stata Corp, College Station, TX, U.S.A.).

## Results

### Included studies

**Figure 1:**
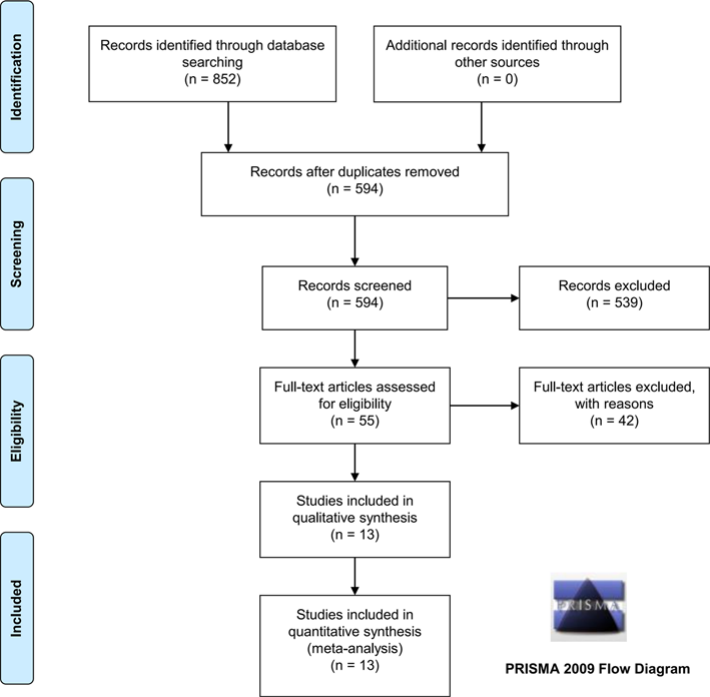
PRISMA-based flowchart of database searching and study selection

We gathered a total of 852 relevant publications from a search of three databases, specifically 201 publications in PubMed, 228 in WOS, and 423 in Embase. Of 852 publications, the following records were excluded: duplicate publications (*n*=258), reviews (*n*=256), cases or trials (*n*=40), cell or animal data (*n*=39), meeting abstract or poster (*n*=64), meta-analysis (*n*=8), polyps that were not colorectal (*n*=76), or the absence of folate, homocysteine, and vitamin B_12_ levels (*n*=56). We then obtained a total of 55 publications with full text for eligibility and removed 31 publications containing the data of intake of folate or vitamin B_12_, and 11 publications that lacked control data or S.D. data. Finally, a total of 13 case–control studies [15,18–29] were enrolled for our quantitative synthesis. We show the inclusion process in Figure 1 and provide detailed characteristics in Table 1. We did not obtain the detailed information of hospital or population-based control (PB) source in several studies, which were recorded as ‘NA’ (Table 1).

**Table 1:** Characteristics of studies included in this meta-analysis

First author	Year	Group	Case			Race	Country	Control			Disease type	Source
			Mean	S.D.	Number			Mean	S.D.	Number		
**Ashktorab et al.** [29]	2007	Serum folate	11.8	4.2	23	Caucasian	U.S.A.	12.7	4	35	Colorectal polyps	HB
		RBC folate	438	140	23	Caucasian	U.S.A.	435	117	35	Colorectal polyps	
		Homocysteine	11.2	3.4	23	Caucasian	U.S.A.	10.9	5	35	Colorectal polyps	
		Vitamin B_12_	561	312	23	Caucasian	U.S.A.	531	265	35	colorectal polyps	
**Beckett et al.** [28]	2015	RBC folate	1.29	0.12	26	Caucasian	Australia	0.93	0.04	118	Female - adenomatous polyps	NA
		RBC folate	0.89	0.07	30	Caucasian	Australia	1.1	0.05	79	Male - adenomatous polyps	
		Vitamin B_12_	0.29	0.02	26	Caucasian	Australia	0.28	0.01	118	Female - adenomatous polyps	
		Vitamin B_12_	0.27	0.02	30	Caucasian	Australia	0.27	13.1	79	Male - adenomatous polyps	
		Homocysteine	12.4	0.79	26	Caucasian	Australia	12	0.4	118	Female - adenomatous polyps	
		Homocysteine	13.8	1.21	30	Caucasian	Australia	13.6	0.52	79	Male - adenomatous polyps	
**Chen et al.** [27]	2014	Homocysteine	12.8	6.6	51	Asian	China	11.2	4.3	99	Colorectal polyps -metabolic syndrome (–)	PB
		Homocysteine	14	6.1	59	Asian	China	11.9	2.9	36	Colorectal polyps-metabolic syndrome (+)	
**Chen et al.**[26]	2013	Homocysteine	14.2	5.5	29	Asian	China	9.8	2.1	96	Adenomatous polyps	PB
		Homocysteine	14.5	7.4	19	Asian	China	9.8	2.1	96	Hyperplastic polyps	
		Serum folate	23.9	17.2	29	Asian	China	19.7	11	96	Adenomatous polyps	
		Serum folate	18.6	9	19	Asian	China	19.7	11	96	Hyperplastic polyps	
		Vitamin B_12_	334	189	29	Asian	China	373	205.4	96	Adenomatous polyps	
		Vitamin B_12_	355	162	19	Asian	China	373	205.4	96	Hyperplastic polyps	
**Chiang et al.** [25]	2015	Homocysteine	13.3	4.94	70	Asian	China	11.6	4.97	182	Adenomatous polyps	PB
		Serum folate	13.3	9.14	70	Asian	China	15.3	8.31	182	Adenomatous polyps	
**Choi et al.** [24]	2015	RBC folate	974	511	37	Caucasian	Australia	1045	576.1	162	Adenomatous polyps	NA
		Homocysteine	9.9	2.9	37	Caucasian	Australia	10	2.6	162	Adenomatous polyps	
**Levine et al.** [23]	2000	RBC folate	261	146	518	Caucasian	U.S.A.	270	153.2	554	Adenomatous polyps	PB
**Lim et al.** [22]	2012	Homocysteine	13.3	3.9	422	Asian	Korea	13.2	5.88	617	Adenomatous polyps	PB
**Lucock et al.** [21]	2011	RBC folate	990	87	38	Caucasian	Australia	914	33	164	Adenomatous polyps	NA
		Serum folate	20.2	1.9	38	Caucasian	Australia	19.5	0.8	164	Adenomatous polyps	
**Lucock et al.** [20]	2015	Homocysteine	13.2	0.73	57	Caucasian	Australia	12.7	0.322	192	Adenomatous polyps	PB
**McGlynn et al.** [15]	2013	RBC folate	474	234	40	Caucasian	Ireland	524	285	53	Adenomatous polyps	HB
		RBC folate	561	290	16	Caucasian	Ireland	524	285	53	Hyperplastic polyps	
		Homocysteine	11.9	5.5	40	Caucasian	Ireland	9.4	2.4	53	Adenomatous polyps	
		Homocysteine	10.2	2.4	16	Caucasian	Ireland	9.4	2.4	53	Hyperplastic polyps	
		Vitamin B_12_	356	162	40	Caucasian	Ireland	383	168	53	Adenomatous polyps	
		Vitamin B_12_	446	184	16	Caucasian	Ireland	383	168	53	Hyperplastic polyps	
**Paspatis et al.** [19]	1995	Serum folate	4.57	2.8	62	Caucasian	Greece	5.09	2.7	50	Adenomatous polyps	HB
		RBC folate	536	273	62	Caucasian	Greece	744	297.1	50	Adenomatous polyps	
**Powers et al.** [18]	2007	Vitamin B_12_	346	214	91	Caucasian	Ireland	311	190.1	85	Colorectal polyps	PB

Abbreviations: HB, hospital-based; NA, not available; PB, population-based.

### Meta-analysis of folate

**Figure 2:**
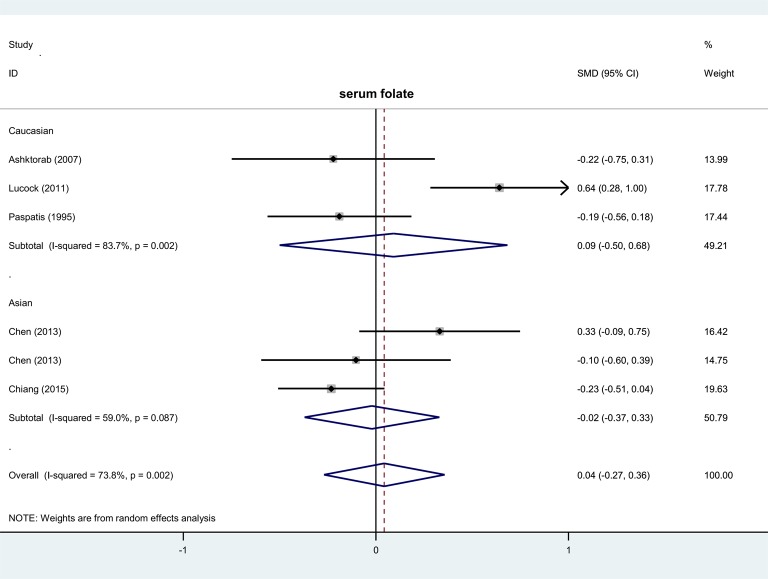
Subgroup analysis of association between serum folate level and risk of colorectal polyps stratified by race

**Figure 3:**
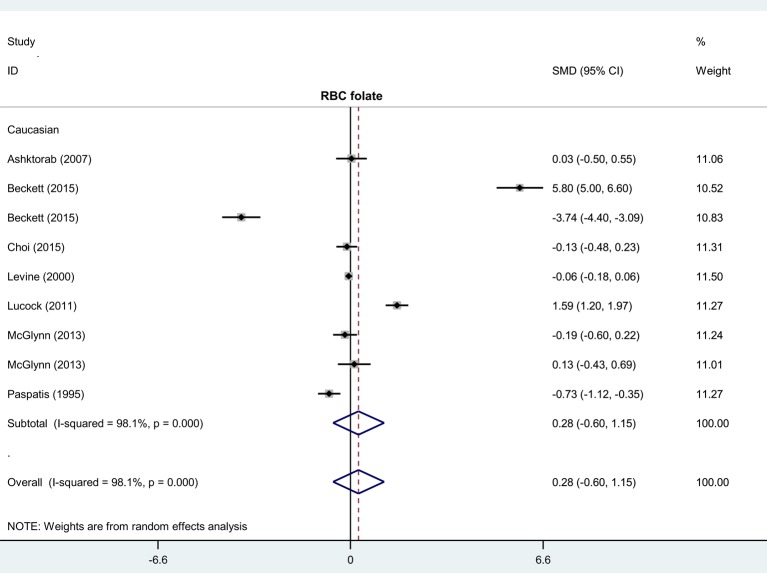
Subgroup analysis of association between RBC folate level and risk of colorectal polyps stratified by race

To evaluate the association between level of serum/RBC folate and risk of colorectal polyps, six case–control studies with 241 cases and 623 controls were enrolled for meta-analysis of serum folate, while 9 case–control studies with 790 cases and 1268 controls were for RBC folate (Table 2). Compared with controls, no increased colorectal polyp risk in cases was detected in the overall meta-analysis (Table 2, *P*>0.05). We also conducted subgroup analyses by country, race, and control source. Similar negative results were obtained (Table 2, all *P*>0.05). It should be noted that we only show the results of subgroup analysis with more than or equal to three case-control studies in the present study. Forest plots of each subgroup analysis by race are given in Figures 2 and 3. These findings suggest that the level of serum or RBC folate seems not to be associated with colorectal polyp risk.

**Table 2:** Meta-analysis of serum folate and RBC folate

Group	Subgroup (factor)*	Sample size		Test of association		
		Studies	Case/control	SMD (95% CIs)	z	*P*-value
**Serum folate**	Overall	6	241/623	0.04 (−0.27, 0.36)	0.28	0.783
	China (country)	3	118/374	−0.02 (−0.37, 0.33)	0.11	0.909
	Caucasian (race)	3	579/753	0.09 (−0.50, 0.68)	0.31	0.760
	Asian (race)	3	118/374	−0.02(−0.37, 0.33)	0.11	0.909
	PB (control source)	3	118/374	−0.02 (−0.37, 0.41)	0.11	0.909
**RBC folate**	Overall	9	790/1268	0.28 (−0.60, 1.15)	0.62	0.536
	Australia (country)	4	131/523	0.87 (−1.89, 3.63)	0.62	0.537
	HB (control source)	4	141/191	−0.22 (−0.62, 0.17)	1.10	0.272
	Caucasian (race)	9	790/1268	0.28 (−0.60, 1.15)	0.62	0.536

Abbreviations: HB, hospital-based; PB, population-based.

* Only the results of subgroup meta-analysis with more than or equal to three case–control studies were provided.

### Meta-analysis of homocysteine, vitamin B_12_

**Figure 4:**
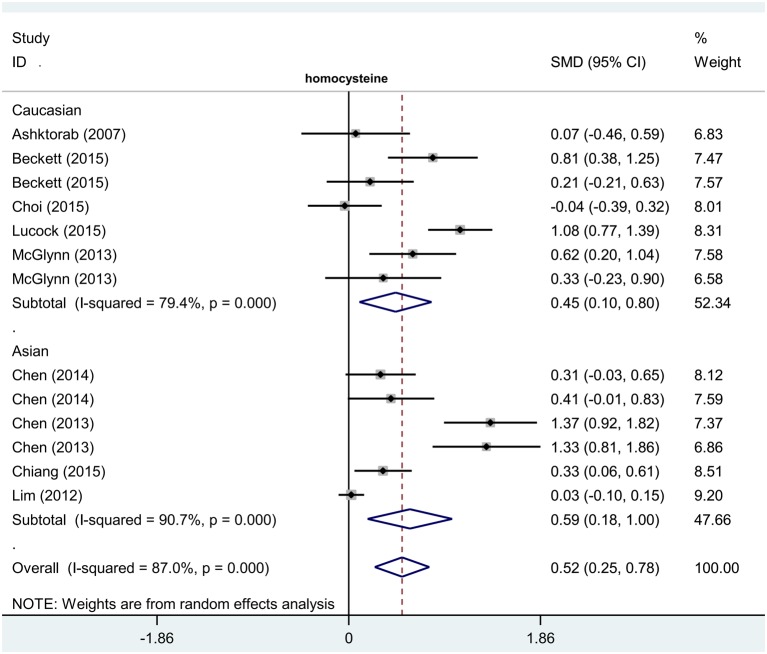
Subgroup analysis of association between homocysteine level and risk of colorectal polyps stratified by race

**Figure 5:**
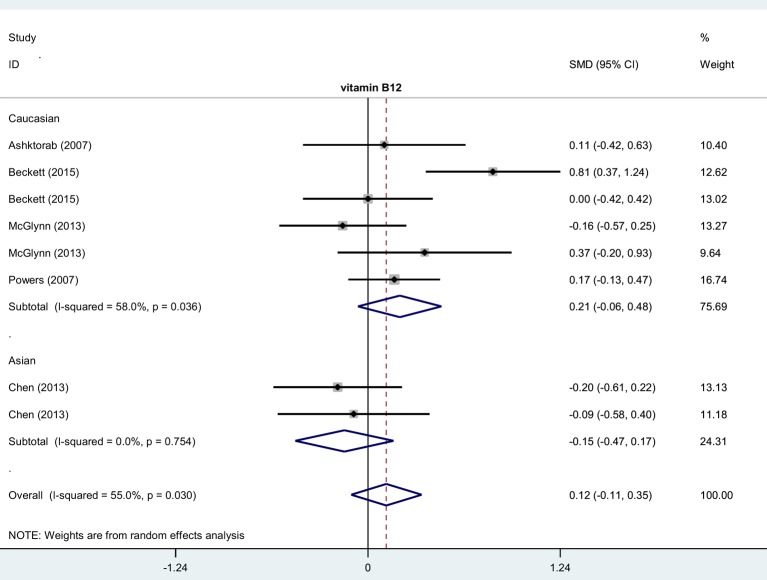
Subgroup analysis of association between vitamin B_12_ level and risk of colorectal polyps stratified by race

Thirteen case–control studies involving 879 cases and 1818 controls were enrolled in this meta-analysis regarding the relationship between the level of homocysteine and colorectal polyp risk. Data from the overall meta-analysis (Table 3) indicated homocysteine level in colorectal polyp cases was higher than in controls free of colorectal polyps (*P*<0.001, SMD = 0.52, 95% CIs = 0.25–0.78). Data from the subgroup analysis of hospital-based control (HB), PB, China, Caucasian, and Asian showed similar positive results (Table 3, all *P*<0.05, SMD > 0). However, no significant difference between cases and controls was observed in the overall or subgroup meta-analyses of vitamin B_12_ (Table 3, all *P*>0.05). Figures 4 and 5 show the forest plots. An elevated level of homocysteine was statistically significantly associated with the risk of colorectal polyps.

**Table 3:** Meta-analysis of homocysteine, vitamin B_12_

Comparison	Subgroup (factor)*	Sample size			Test of association	
		Studies	Case/control	SMD (95% CIs)	*z*	*P*
**Homocysteine**	Overall	13	879/1818	0.52 (0.25, 0.78)	3.84	<0.001
	HB (control source)	3	79/141	0.37 (0.05, 0.70)	2.23	0.026
	PB (control source)	7	707/1318	0.67 (0.26, 1.08)	3.17	0.002
	Australia (country)	4	150/551	0.52 (−0.03, 1.07)	1.86	0.064
	China (country)	5	228/509	0.72 (0.28, 1.16)	3.21	0.001
	Caucasian (race)	7	229/692	0.45 (0.10, 0.80)	2.54	0.011
	Asian (race)	6	650/1126	0.59 (0.18, 1.00)	2.83	0.005
**Vitamin B_12_**	Overall	8	274/615	0.12 (−0.11, 0.35)	1.03	0.305
	HB (control source)	3	79/141	0.05 (−0.25, 0.36)	0.35	0.729
	PB (control source)	3	139/277	0.01 (−0.22, 0.24)	0.11	0.912
	Ireland (country)	3	147/191	0.10 (−0.16, 0.37)	0.76	0.449
	Caucasian (race)	6	226/423	0.21 (−0.06, 0.48)	1.49	0.135

Abbreviations: HB, hospital-based; PB, population-based.

* Only the results of subgroup meta-analysis with more than or equal to three case–control studies were provided.

### Heterogeneity, bias, and sensitivity analysis

**Figure 6:**
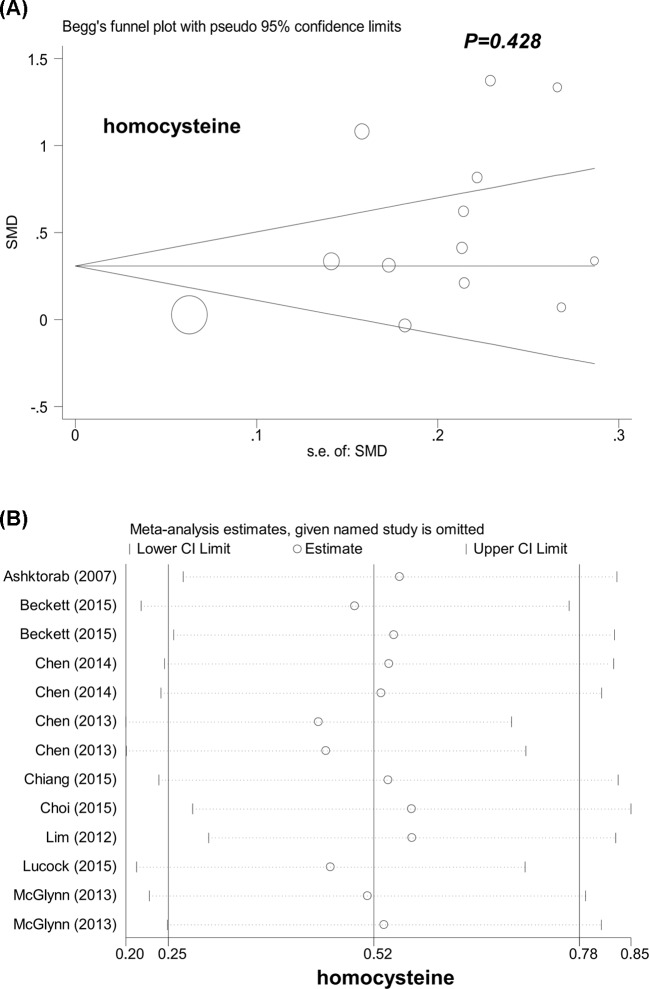
Begg’s funnel plot and sensitivity analysis for homocysteine level and risk of colorectal polyps (**A**) Begg’s funnel plot; (**B**) sensitivity analysis.

Obvious heterogeneity was detected in all the comparisons given above (Table 4, all *I^2^* > 50.0%, *P*-value of heterogeneity <0.05), and IV-weighted random effect models were thus used in Cohen statistics. For publication bias, as shown in Table 4, apart from Egger’s test of homocysteine (*P*=0.024), *P*-value of Begg’s test and Egger’s test was larger than 0.05 in others, indicating the absence of large publication bias. Begg’s funnel plot for the association of homocysteine level and colorectal polyp risks is shown in Figure 6A (homocysteine), and Supplementary Figures S1A (serum folate), S2A (RBC folate), S3A (vitamin B_12_). In addition, similar pooled ORs were observed in our sensitivity analysis, as shown in Figure 6B (homocysteine), Supplementary Figures S1B (serum folate), S2B (RBC folate), and S3B (vitamin B_12_).

**Table 4:** Assessment of heterogeneity and publication bias

Group	*I^2^*	*P*-value	Model	Begg’s test		Egger’s test	
				*z*	*P*	T	*P*
**Serum folate**	73.8%	0.002	Random	0.00	1.000	0.23	0.831
**RBC folate**	98.1%	<0.001	Random	0.94	0.348	0.39	0.707
**Homocysteine**	87.0%	<0.001	Random	0.79	0.428	2.61	0.024
**Vitamin B_12_**	55.0%	0.030	Random	0.87	0.386	0.10	0.921

## Discussion

Because of the important role of methyl group donors, various studies investigated the role of folate, homocysteine, vitamin B_12_ in several clinical diseases. However, no final conclusion was drawn. In 2016, Cao et al. [34] conducted a meta-analysis of 20 eligible studies and found that lower folate level may be related to the risk of schizophrenia. In 2017, the meta-analysis data reported by Wang et al. [35] showed the correlation between reduced serum levels of folate and vitamin B_12_ and the risk of type 2 diabetes mellitus in a Chinese population. The low level of folate was also reported to be linked to the risk of inflammatory bowel disease but that of vitamin B_12_ was not [36]. Ramanujam et al. [37] performed another meta-analysis and found that low folate levels were not statistically significantly associated with the risk of depression.

With regard to CRC, several meta-analyses without consistent conclusions [38–40] explored the association between folic acid supplementation or folate intake and the risk of CRC. A meta-analysis by Kennedy et al. [38] in 2011 showed that higher folate intake level was important for the reduced risk of CRC. However, the negative correlation between folate supplementation and the risk of CRC was also reported in another meta-analysis in 2015 [40]. Colorectal polyps were considered precursors of CRC. Nevertheless, we failed to observe the relevant meta-analysis for the effect of folate, homocysteine, vitamin B_12_ in the risk of colorectal polyps. We also observed the different reports in distinct populations. For instance, the high level of folate was reported to be associated with the increased risk of CRC patients with adenomatous polyps, but not in CRC patients without adenomatous polyps in a Chinese population [25]. The evaluated plasma homocysteine level was associated with an increased susceptibility to colorectal polyps in Chinese population [26]. However, no relationship was found between the level of serum folate, RBC folate, vitamin B_12_, or homocysteine and risk of colon polyps in African Americans [29]. We were, therefore, very interested in enrolling all the published articles to assess this relationship.

According to our strict searching and screening requirements, a total of 13 eligible case–control studies containing data covering sample size, mean value, and S.D. were enrolled. Our findings showed that the level of homocysteine in colorectal patients with polyps was significantly higher than that in controls without polyps. In contrast, the level of serum folate, RBC folate, and vitamin B_12_ did not differ between polyp patients and controls.

Several limitations should be fully considered. (i) As in other meta-analyses, there exists the problem of limited case–control studies for quantitative synthesis. Only one study provided data covering the association between serum vitamin B_12_ level and colorectal polyps in an Asian population [26], which caused the subgroup analysis of Asians for vitamin B_12_ to fail. Here, we only provided the data of subgroup meta-analysis using such factors as country, race, and control source when there were three or more case–control studies included. Additionally, although no statistical effect was observed the level of serum folate, RBC folate, and vitamin B_12_, we still cannot disregard their potential influence in the progression of colorectal polyps yet because of the limited sample size. (ii) A higher plasma homocysteine concentration was reported to be associated with the risk of advanced adenoma in female Korean participants, but not in the overall population [22]. The influence of folate supplementation on changes in the number of recurrent polyps was also reported [41]. For this reason, we performed further detailed subgroup analyses by specific disease type, gender, should be performed, whenever we were able to acquire more data. More biochemical variables, such as plasma riboflavin (nmol/l) and plasma flavinsk (nmol/l), should be investigated as well. (iii) Our meta-analysis showed considerable heterogeneity. We note that the control groups of some of the studies included here were hospital-based and, in some cases, relevant information was unavailable. We did not observe any obvious decrease in heterogeneity in our stratified meta-analyses (data not shown). Insufficient data may prevent successful identification of the cause of heterogeneity. (iv) Slight publication bias was observed in the Egger’s test of homocysteine, which may reduce the statistical power with respect to the positive correlation between homocysteine level and colorectal polyp susceptibility to some extent.

Data regarding several studies support the association between the high homocysteine concentrations in the risk of developing CRC [42,43]. However, the specific mechanism underlying the role of an increased homocysteine level in susceptibility to colorectal polyps remains elusive. Folate metabolism is dependent on 5,10-methylenetetrahydrofolate (MTHFR) enzyme, which catalyzes the conversion of MTHFR into 5-methyltetrahydrofolate (5-methyl THF) [14]. Vitamin B_12_ is also essential to the conversion of homocysteine into methionine [14]. Homozygous and heterozygous mutations of the *MTHFR* gene for C677T polymorphism have been reported to render enzyme activity lower than in the wild-type genotype [14,17]. We should also consider more factors, such as genomic instability, DNA synthesis, CpG sequences methylation, and other epigenetic changes.

## Conclusion

Generally speaking, quantitative synthesis data provide evidence regarding the role of an elevated homocysteine level in the developing colorectal polyp risk. Larger sample sizes were still required to investigate whether serum folate, RBC folate, and vitamin B_12_ levels function in the susceptibility to colorectal polyps.

## Supporting information

**Figure S1:** 

**Figure S2:** 

**Figure S3:** 

## Abbreviations

CI: confidence interval
CRC: colorectal cancer
Embase: Excerpta Medica Database
IV: inverse variance
MTHFR: 5,10-methylenetetrahydrofolate
PB: population-based control
RBC: red blood cell
SMD: standard mean difference
WOS: Web of Science
