# Supplementary material for: Colorectal polyp risk is linked to an elevated level of homocysteine
Source: Biosci Rep. 2018 Apr 20;38(2):BSR20171699. doi: 10.1042/BSR20171699 (PMC5968185; doi:10.1042/BSR20171699)
Supplement: Supplementary file 1 [file bsr20171699_Supp1.pdf]

Figure S1

A

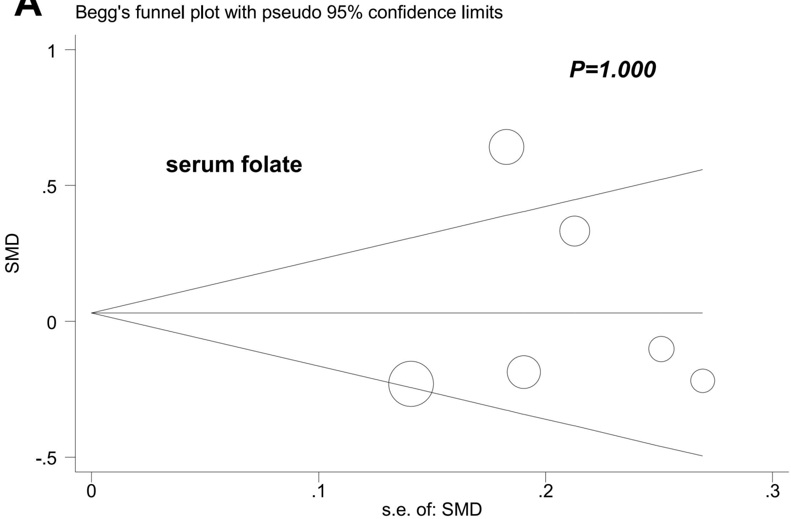
Begg's funnel plot with pseudo 95% confidence limits
P=1.000
serum folate
SMD
1
.5
0
-.5
0
.1
.2
.3
s.e. of: SMD

B

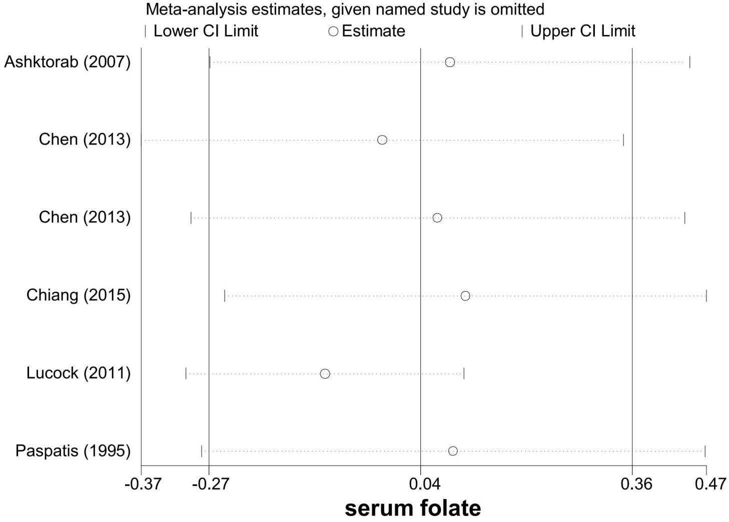
Meta-analysis estimates, given named study is omitted
Lower CI Limit
Estimate
Upper CI Limit
Ashktorab (2007)
Chen (2013)
Chen (2013)
Chiang (2015)
Lucock (2011)
Paspatis (1995)
-0.37
-0.27
0.04
0.36
0.47
serum folate

Figure S2

A

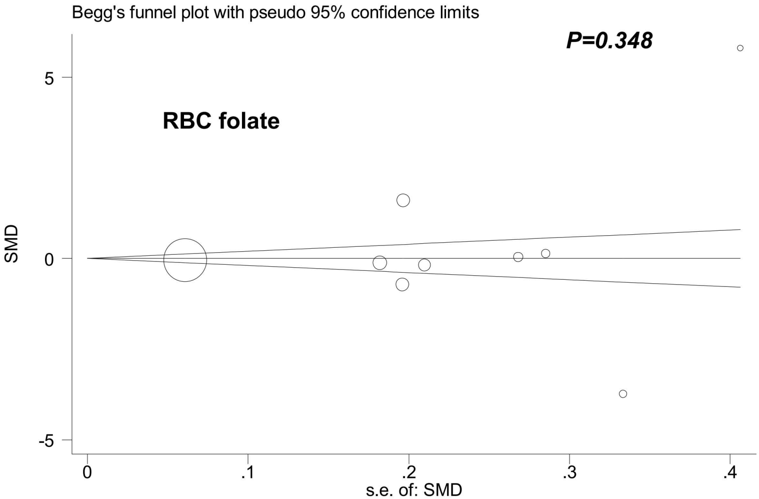

Begg's funnel plot with pseudo 95% confidence limits
P=0.348
RBC folate
SMD
5
0
-5
0
.1
.2
.3
.4
s.e. of: SMD

B

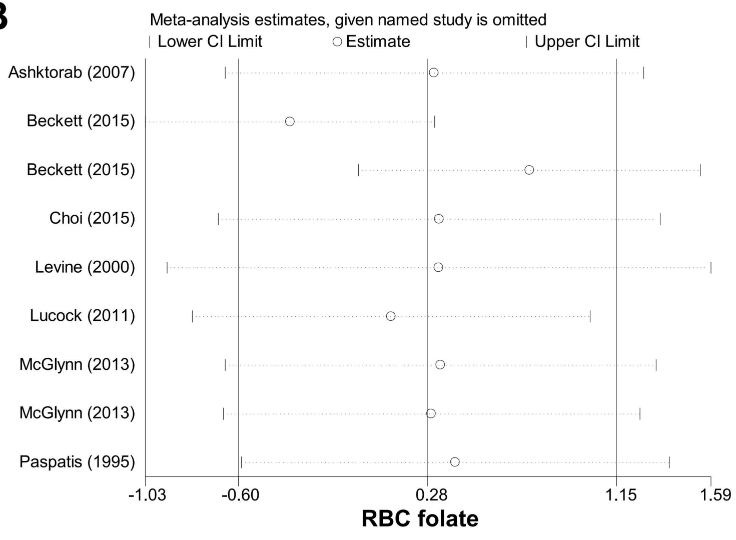

Meta-analysis estimates, given named study is omitted
Lower CI Limit
Estimate
Upper CI Limit
Ashktorab (2007)
Beckett (2015)
Beckett (2015)
Choi (2015)
Levine (2000)
Lucock (2011)
McGlynn (2013)
McGlynn (2013)
Paspatis (1995)
-1.03
-0.60
0.28
1.15
1.59
RBC folate

Figure S3

A

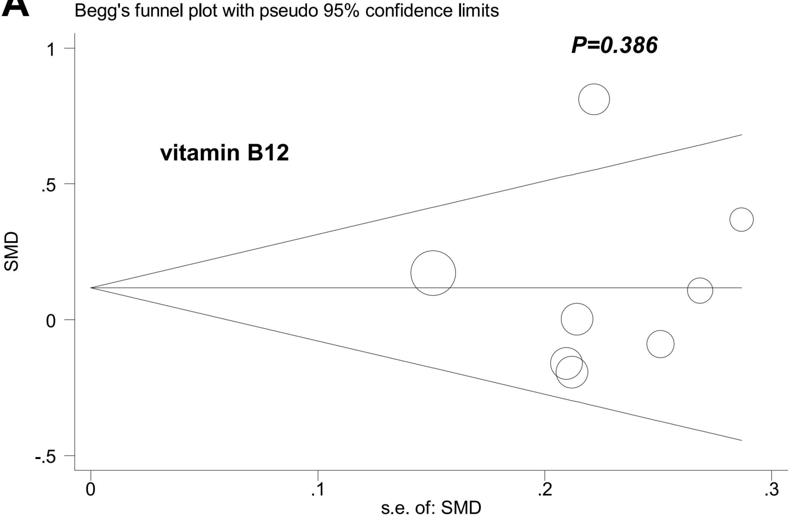
Begg's funnel plot with pseudo 95% confidence limits
P=0.386
vitamin B12
SMD
1
.5
0
-.5
0
.1
.2
.3
s.e. of: SMD

B

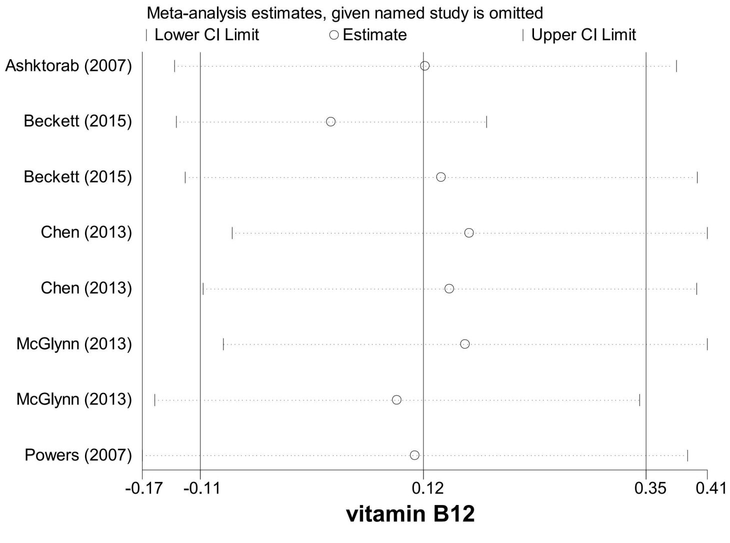
Meta-analysis estimates, given named study is omitted
Lower CI Limit
Estimate
Upper CI Limit
Ashktorab (2007)
Beckett (2015)
Beckett (2015)
Chen (2013)
Chen (2013)
McGlynn (2013)
McGlynn (2013)
Powers (2007)
-0.17
-0.11
0.12
0.35
0.41
vitamin B12
